# Supplementary material for: Strengthening clinical bacteriology laboratory diagnostics to combat sepsis and antimicrobial resistance in Benin: a train-the-trainer approach
Source: Front Med (Lausanne). 2024 Apr 19;11:1281418. doi: 10.3389/fmed.2024.1281418 (PMC11066218; doi:10.3389/fmed.2024.1281418)
Supplement: Supplementary file 1 [file Table_1.DOCX]

**Strengthening Clinical Bacteriology Laboratory Diagnostics to combat Sepsis and Antimicrobial Resistance in Benin: A Train-the-Trainer Approach**

Hornel Koudokpon^1^, Brice Boris Legba^1^, Victorien Dougnon^1*^, Sointu Mero^2,3^, Honoré Bankole^1^, Kaisa Haukka^3,4^

^1^Research Unit in Applied Microbiology and Pharmacology of Natural Substances, Research Laboratory in Applied Biology, Polytechnic School of Abomey-Calavi, University of Abomey-Calavi, Abomey-Calavi, Benin

^2^Human Microbiome Research Program, Faculty of Medicine, University of Helsinki, Helsinki, Finland

^3^Physicians for Social Responsibility, Helsinki, Finland

^4^Department of Microbiology, University of Helsinki, Helsinki, Finland

***Correspondence:**

**Victorien Dougnon**

**[Victorien.dougnon@gmail.com](mailto:Victorien.dougnon@gmail.com)**

**Abstract**

Improved laboratory diagnostics is needed to support sepsis diagnosis and combat increasing antibiotic resistance in Benin. We trained clinical laboratory experts and technicians to improve their skills in accurate and up-to-date diagnostics. A Train-the-Trainer (TtT) approach was used to design the course that combines theoretical and practical laboratory skills, specifically addressing the knowledge gaps we had previously identified in our national survey. Pedagogical methods were student-centered, including peer learning, use of online materials, practical laboratory work and pre- and post-course tests. We first trained 10 trainers who in turn trained 40 laboratory technicians from across the country, from both public and private clinical and veterinary laboratories. The trainers also prepared standard operation procedures for blood culture and antibiotic susceptibility testing based on international standards. Three months after the training, follow-up visits were made to the laboratories where the implementation of the new skills was evaluated. The progress of the participants observed during the course and the implementation of the new skills afterwards proved the training to be effective. The professional network s created during the training, the empowerment that utilizes local knowledge resources, and the government support for our initiative can be expected to bring sustainability to the initiative and support the participation of Beninese laboratories in international surveillance programs in the future.

**Keywords:** Train-the-Trainers, clinical bacteriology diagnostics, sepsis, antibiotic susceptibility testing (AST), Benin

1. **Introduction**

The clinical bacteriology laboratory plays a key role as a provider of diagnostic services for quality patient care Blood cultures and Antibiotic Susceptibility Testing (AST) help identify the cause of sepsis and determine antimicrobial resistance (AMR) of the pathogens (1) . Sepsis is life-threatening organ dysfunction caused by a dysregulated host response to infection (2). In 2017, 11 million deaths were reported to be sepsis-related, accounting for approximately 20% of all global deaths (3). In 2019, the global impact of AMR was estimated to be 4.95 million deaths due to infections caused by resistant bacteria (4). Neonatal sepsis is the third leading cause of neonatal death worldwide, particularly in low- and middle-income countries (LMICs), with many of the deaths attributed to AMR (5 - 8). Africa is the most affected continent where many endogenous factors and the increasing prevalence of AMR complicate the treatment of sepsis (6, 9 – 11). Furthermore, reliable diagnostic laboratory testing is not readily accessible in sub-Saharan Africa, leading to frequent misdiagnosis (10, 12 – 13).

A recent study by Legba et al. (14) in Benin reported that six laboratories in the country can perform blood cultures, while AST is done in 23 laboratories. However, the number of tested samples is very low due to inadequate infrastructure and costs to a patient. Additionally, deficient skills of laboratory personnel lead to unreliable test results. Therefore, nationally uniform training based on standardized techniques is needed to improve the accuracy of laboratory diagnostics (12, 15). Reliable test results are essential for both targeted treatment of patients and empiric treatment when the prevalent AMR characteristics of the sepsis-causing organisms are known in the region (14).

To improve the quality of sepsis diagnostics and AST in all regions of Benin, we adopted a Train-the-Trainer (TtT) approach to train several laboratory professionals in a short time and ensure sustainable transfer of new skills. TtT is a training methodology where individuals, experienced in a particular subject or skill, undergo training to become trainers themselves. The goal is to empower these individuals to deliver the same training to others, creating a cascading effect of knowledge dissemination (16). The TtT approach has been successful in similar continuous professional training initiatives in LMICs (17 - 20). In our Beninese setting, the TtT approach was considered new and innovative, since primarily instructor-centered teaching methods, such as lecturing to large classes, are used and resources for hands-on laboratory training are scarce. The traditional approach does not provide the kind of skills that technicians need in their work, nor does it allow for monitoring the practical application of the concepts learned in training. Therefore, we chose methods that encourage peer-learning to enable experienced technicians to both update their skills and to pass on their knowledge to others, without compromising the quality of teaching.

Our training initiative was aligned with the governmental goal to improve laboratory functioning and reliability in Benin (21). The Beninese national action plan against Antimicrobial Resistance (PAN-RAM –Bénin 2019 -2024), strategic axis 2 (Surveillance, laboratory capacity and research on AMR) provides guidelines for the continuous training of laboratory personnel working in microbiological laboratories (Action 2.2.1.1) and networking of laboratories for the quality approach. Accordingly, the objective of our training was to build a network of trainers with good skills and capable of training more laboratory technicians to improve the quality of clinical bacteriology laboratory diagnostics in Benin.

1. **Methods, implementation, and results**
   1. **Identification of the training needs**

Before starting the training activities, a survey was conducted using face-to-face interviews with laboratory staff and physicians across the country to assess the training needs (14). The survey identified deficiencies in the knowledge and practices of laboratory technicians regarding culturing blood samples, identifying pathogens causing sepsis and AST. The survey, along with additional visits to selected hospitals in different parts of the country and all the physicians interviewed, indicated dissatisfaction with the local laboratory services, the laboratory equipment used for AST, the proficiency of the laboratory staff and reliability of the results. The laboratory technicians and managers as well as the hospital management strongly supported the planned training activities. The Ministry for Health of Benin also endorsed our training activity, which is crucial for ensuring the initiative's sustainability.

The flowchart for the training is shown in Figure 1. To develop the course curriculum, eight experts consisting of Beninese and Finnish laboratory and health professionals were involved in formulating the course objectives and methods. Four topics were chosen for the training: 1) blood culture; 2) bacterial identification techniques; 3) AST; and 4) quality assurance in laboratory.

- 1. **Recruitment of the trainers and trainees**

Ten trainers to enroll were identified based on their academic qualifications. They were medical microbiologists holding at least a Master's degree and a position as a senior technician in a hospital bacteriological laboratory or they were involved in University training in medical microbiology as teachers or PhD students. Further 40 technicians selected as trainees included 25 technicians from the laboratories carrying out bacteriological examinations including AST. Further 13 technicians were chosen from among laboratory technicians working at the universities as research laboratory technicians. Benin has only two veterinary laboratories, and both were asked to send a laboratory technician to the course, which they did. Of the 40 technicians participating the training, 22 were males and 18 females. 24 technicians were aged 20-30 and 16 aged 31-50.

- 1. **Course development as a way to train the trainers**

In developing the training course, a learner-centered participatory method was used, with trainers and experts collaboratively designing the content and materials. The three-week TtT course included individual and group study, discussions, laboratory practice and exchange of best practices among participants.. Through sessions of meticulous discussions to reach consensus, they also created a comprehensive guide for a new five-day course. This guide includes 21 standard operating procedures (SOPs) for bacteriological laboratories, aligned with WHO guidelines but adapted to local conditions and validated by Beninese and Finnish clinical microbiology professionals.

- 1. **Structure for the five-day training course**

The course for the trainees covered four topics over 5 days (40 hours), followed by a shorter sixth day dedicated to the final assessments and closing ceremony. The topics were, , Days 1 and 2: conducting a blood culture using manual method, following instructions of WHO Laboratory Manual for the Examination and Processing of Human Blood, Day 3: Bacterial identification using API and biochemical tests for *Staphylococcus aureus* identification, Day 4: conducting AST using disk diffusion method following the EUCAST guidelines, Day 5: internal and external quality assurance in bacteriological laboratory according to the WHO recommendations. Each day was structured to contain introduction to the topic by the trainers, independent work, group work, practical laboratory work and recapping the learned contents together with the trainers. The detailed program for the training is shown in Table 1. Altogether 40 trainees participated the three training courses, each including 13-14 technicians.

- 1. **Learning philosophy and teaching approaches**

In planning the pedagogical approach, we were inspired by the principal of the constructivist learning theory: “Constructivism implies that learners are encouraged to construct their own knowledge instead of copying it from an authority, be it a book or a teacher, in realistic situations instead of decontextualized, formal situations such as propagated in traditional textbooks, and together with others instead of on their own (22 - 23). The TtT approach aligns well with this pedagogical principal as it promotes learner engagement, reflective practice, critical thinking, and skills development.

The specific pedagogical techniques we applied included:

Pre-assignments. A few months before the training, the participants were requested to follow the Massive Open Online Courses (MOOCs) listed in Table 2. At the beginning of the training, the themes of the online courses were discussed with the participants, allowing us to assess what they had learnt.

***Peer learning.*** Pairing a more experienced and a more novice technician to work together in the laboratory and during various fact-finding activities created an opportunity to pass on practical experience and to develop the teaching skills of the trainers and trainees.

***Utilizing freely available online audiovisual and reading materials.*** The trainers/trainees searched information from sources such the YouTube videos produced by reliable authorities in specific topics. Some web sites were indicated by the experts, but the trainers and trainees were also encouraged to search for new sites, eg. the major French universities and hospitals provide suitable material in French. For future sustainable continuous independent learning, it was important for the trainers and trainees to get familiar with using freely available up-to-date material, since the instructions available in the Beninese clinical laboratories are often outdated and the chances to participate professional further education are rare. Materials utilized on the course are listed in Table 2.

***Laboratory work.*** The 5-day training course took place primarily in the laboratory allowing the trainees to put in practice what they had learnt, for example from the YouTube videos. This was exceptional in the resource-poor settings, where the financing is seldom enough for providing all the learners an opportunity to hands-on experimentation. In addition, some role-play components were included, such as the pairs taking a blood sample from each other under the guidance of a qualified nurse. The blood samples were spiked with *Escherichia coli, Klebsiella pneumoniae, Salmonella spp., Staphylococcus aureus* or a mixture of *E. coli* and *S. aureus* by the instructors, without the course participants knowing the identity of the bacteria. During the training the participants practiced identifying the bacteria in their own samples and conducting AST to them.

- 1. **Evaluation of the training results and methods**
     1. ***On-site assessment by pre- and post-tests***

At the beginning of the onsite training, the participants' knowledge and skills on the training topics were assessed by pre-tests including both theoretical questions and practical laboratory tasks (Appendix ). The theoretical part of the test included open questions on the concept of sepsis, indicators for blood culture contamination, identification of typical bacteria infecting blood, choosing antibiotics for AST and quality control. For the practical part in the laboratory, each participant was given a sample containing *E. coli*, *S. aureus*, both, or neither in Müller-Hinton broth. The participant, not knowing the bacterial content of the broth tube, carried out the macroscopic and microscopic examination of the sample, chose the appropriate solid culture medium and carried out the inoculation.

The pre-test assessment allowed some last-minute adjustments in the course contents to meet the specific knowledge gaps of the participants. It also served as self-assessment for the trainees. The pre- and post-tests were identical allowing us to evaluate the improvement in the participants’ knowledge and skills during the course and efficacy of the training. To measure the progress, we compared participants’ test results before and after the training (Table 3). The average improvement of the test score was 4.5 and the p-value 0.001 indicating indicated a significant difference between the scores obtained by the participants before and after the training.)

All the pairs were also asked to produce a course report that summarized the practical work carried out, including the methodology and the results obtained. This allowed the instructors to evaluate the pair-work and learning outcomes. The participants were also asked to list the three most important concepts or skills that they had learned during the training. The answers included items such as: taking a blood sample for a blood culture, interpreting the API20E results, conducting, interpreting, and controlling the quality of AST, phenotypic techniques for bacterial identification, familiarization with the latest version of EUCAST breakpoints set by the European Committee on Antimicrobial Susceptibility Testing.

- - 1. ***Feedback on the training by the trainees and trainers***

Feedback forms completed by participants at the end of the training showed unanimous approval of the teaching methods, with excellent or very good ratings (Appendix 1). Participants were also satisfied with the organization of the training, logistics, atmosphere, time management and the competence of the trainers. In the open feedback, the innovative teaching approach and small group sizes were particularly appreciated, because they allowed adjustments to course structure and individualized support. The trainers, in turn, expressed their satisfaction with the high motivation of the participants, the benefits of the new teaching approach and the encouraging working environment created by small group sizes. They recommended frequent offering of similar courses. In the future, including new bacterial species (eg. *Heamophilus influenzae* and *Streptococcus* spp.) was considered to be important as well as allocating even more time for practical exercises.

- - 1. ***Post-training on-site monitoring***

The trainees were expected to apply and communicate the newly acquired knowledge in their home laboratories and to train their colleagues. Three months after the course, an on-site evaluation was carried out by visiting 15 of the laboratories to assess the improvements to the daily practice after the training. The evaluation was carried out in the actual work situation and lasted four hours in each laboratory. We had previously collected baseline information from the laboratories (14), which allowed us to assess the positive changes in the practices related to external and internal quality assurance, a blood culture, bacterial identification methods and AST. During the visits, the evaluators noticed that the new SOPs produced by the trainers were in effective use. However, shortcomings were detected, for example, in sample handling and storage, storage of quality control strains and interpretation of AST results and the technicians were advised for necessary corrections. Consequently, managers of the health establishments (physicians in most cases) expressed their satisfaction to the training results.

1. **Discussion**

We designed and implemented a Train-the-Trainer initiative aimed at improving the quality of clinical bacteriology laboratory diagnostics for bacteria causing sepsis and AST. We first trained 10 laboratory professionals to be trainers, who then carried out training of 40 trainees, who were laboratory technicians from different parts of Benin. The TtT approach was chosen, since it is suited for short continuous professional training needs, can combine expertise of international and local experts, can be completed in a short time, creates a cascade of knowledge transfer and is scalable. Our training improved the skills of the Beninese laboratory technicians. They can provide more reliable information for effective treatment of patients and data for monitoring pathogens causing blood infections and their AMR provide. We were able to promote standardization of the laboratory testing techniques in Benin by inviting participants from different regions and sharing with them the SOPs drafted jointly by the experts and trainers. By basing the SOPs on the WHO recommendations, our purpose is to enhance reporting of the Beninese laboratory results to international surveillance systems such as the Global Antimicrobial Resistance and Use Surveillance System (GLASS).

The central government has previously run some training courses for clinical bacteriology laboratories, but ours were the first to involve both the private and public laboratories to harmonize the national practices in line with the international standards. The national strategy for fighting AMR emphasizes the One Health approach (21), but in practice the laboratory services for the animal and environmental samples are even more lacking than for the human samples. Also, the One Health aspect was considered by including technicians from the only two veterinary laboratories in Benin into the training activities. We included in the training technicians from both clinical and veterinary laboratories to enhance adopting similar laboratory techniques for human, animal and environmental samples and consequently allowing, for example, easier tracing of infection sources.

Typically, access to continuous training is restricted in LMICs, and factors such as the poor fit between existing learning activities and the desire of participants for practical learning, and uninspiring teachers can reduce motivation to attend available training (24). Our interactive, learner-centered methods were able to circumvent these problems. Feedback discussions during training of the trainers showed clearly that networking with colleagues and writing the SOPs were seen to be very empowering to the participants. Many laboratory professionals, especially the senior ones, who have collected plenty of valuable silent knowledge during their career, stated that this was the first time they were heard as real experts in laboratory diagnostics. They considered keeping up the newly formed collegial network as important way to share up-to-date knowledge and concerns also in the future. Together they can also have a stronger influence in planning the laboratory diagnostic practices in the hospitals.

We monitored the impact of training by making follow-up visits to the laboratories. Ideally the visits would be repeated after some time, for example half a year after the training, to ensure the positive long-term effects of the training Changes such as job transfers can compromise the gains of the training and for sustainable improvement, it is essential to organize the training courses in regular bases so that more staff from each laboratory would have access to them. Also formalizing the peer-mentoring as part of a formal apprenticeship program for new laboratory technicians would be a way to perpetuate the achievements. The course can also serve as a basis for the development of a more elaborate continuing education programme supported by the Ministry of Health..

In this communication, we have highlighted the pedagogical choices made, the objectives achieved, and the feedback from the participants to our bacteriology laboratory diagnostics training. We hope that our experience can serve as a model that inspires the creation of more TtT activities for continuous training of professionals in various fields in Benin and beyond. For sustainability of the activity, the continuous endorsement by the local Ministry of Health or another relevant ministry is needed. In our case, the initial plan involved the presence of Finnish clinical laboratory experts to participate the training, but due to the travel restrictions caused the Covid19 epidemic, the training was organized utilizing local expertise in Benin, with the foreign experts mainly commenting the written plans for training and giving some online support. The new approach was saluted with enthusiasm among the Beninese professionals and many previously hidden skill resources were successfully tapped. The approach based on the active participation of local experts to guide continuous education and to encourage methods that actively engage trainers and trainees was new in Benin.It turned out to be very successful and empowered the local healthcare experts and laboratory professionals.

1. **Conclusions**

The Train-the-Trainer approach was shown to be an effective way to enhance the skills of laboratory professionals. Our training initiative combined theoretical and practical learning to improve the skills and to correct knowledge gaps identified during a national survey. It enabled experienced technicians to not only update their skills in practices related to blood culture and AST, but also to network and share their knowledge with others, promoting sustainable transmission of the new skills. Course training was combined with the development of standardized SOPs adapted to local conditions. The SOPs were implemented in local laboratories with the help of the trainees, and their use was examined during follow-up visits to the laboratories. This will contribute to improve the accuracy and reliability of diagnostic test results in the clinical bacteriology laboratories in Benin. Our training initiative represents a successful strategy for strengthening laboratory capacity in resource-limited settings. The recommendations arising from the training, such as extending training to a larger number of laboratory professionals, will be duly considered for inclusion in future projects.

**Data availability statement**

The course material is available through the link provided in Appendix 3.

**List of appendices**

Appendix 1: Assessment of the training by the participants

Appendix 2: Pre-test/post- test

Appendix 3: Course material

**Ethics statement**

Ethical review and approval were not required for the study in accordance with the local legislation and institutional requirements.

**Consent statement**

Written informed consent to participate in this study was provided by the participants.

**Author contributions**

BBL, VD, KH contributed to the conception and design of the training. HK, BBL, VD, KH, SM, HB implemented the training and collected the data. SM verified the SOP's used for the training. HK, BBL, VD, KH wrote the manuscript. SM, HB commented on the manuscript. All authors contributed to the article and approved the submitted version.

**Acknowledgments**

We are grateful to our trainers, who showed great devotion and enthusiasm in developing the course contents and materials with us. We thank the Beninese experts and the Finnish professional volunteers for their expert advice to improve the quality of laboratory diagnostics in Benin. The initiative was funded by the grant from the Finnish Ministry for Foreign Affairs to the Physicians for Social Responsibility (PSR) - Finland for the “Quality laboratory testing” training project in Benin.

**Conflict of interest:**

The authors declare that there are no competing interests.

**References**

1. Park, J-M., Kwon, M., Hong, K.H., Lee, H., Yong, D. (2023). European Committee on Antimicrobial Susceptibility Testing-Recommended Rapid Antimicrobial Susceptibility Testing of Escherichia coli, Klebsiella pneumoniae, and Staphylococcus aureus From Positive Blood Culture Bottles. Ann Lab Med. 2023;43:443–50.
2. Singer, M., Deutschman, C.S., Seymour, C.W., Shankar-Hari, M., Annane, D., Bauer, M., et al. (2016). The Third International Consensus Definitions for Sepsis and Septic Shock (Sepsis-3). JAMA, 315:801–10. https://doi.org/10.1001%2Fjama.2016.0287
3. Rudd, KE., Johnson, SC., Agesa, KM., Shackelford, KA., Tsoi, D., Kievlan, DR., et al. (2020). Global, regional, and national sepsis incidence and mortality, 1990-2017: analysis for the Global Burden of Disease Study. Lancet 395:200–211. https://doi.org/10.1016/S0140-6736(19)32989-7
4. GBD Antimicrobial Resistance Collaborators. (2019). Global mortality associated with 33 bacterial pathogens in 2019: a systematic analysis for the Global Burden of Disease Study 2019. Lancet.;400(10369):2221-2248. doi: 10.1016/S0140-6736(22)02185-7. Epub
5. Do, SN., Luong, CQ., Pham, DT., Nguyen, MH., Nguyen, NT., Huynh, DQ. et al. (2021). Factors relating to mortality in septic patients in Vietnamese intensive care units from a subgroup analysis of MOSAICS II study. Sci Rep. 11:18924. https://doi.org/10.1038/s41598-021-98165-8
6. Pillay, D., Naidoo, L., Swe-Han, K. and Mahabeer, Y. (2021). Neonatal sepsis in a tertiary unit in South Africa. BMC Infect Dis 21(1):225
7. Milton, R., Gillespie, D., Dyer, C., Taiyari, K., Carvalho, M., Thomson, K. et al. (2022). Neonatal sepsis and mortality in low-income and middle-income countries from a facility-based birth cohort: an international multisite prospective observational study. Lancet Glob. Health. 10:e661–e672. https://doi.org/10.1016/S2214-109X(22)00043-2
8. Wang, J., Zhang, H., Yan, J. and Zhang, T. (2022). Literature review on the distribution charateristics and antimicrobial resistance of bacterial pathogens in sepsis. J Matern Fetal Neonatal Med. 35(5):861-870. Doi: 10.1080/14767058.2020.1732342.
9. O'Neill, J. (2016). Tackling drug-resistant infections globally: final report and recommendations. Review of Antimicrobial resistance. Wellcome Trust and HM Government. 84p.
10. Ouedraogo, AS., Jean Pierre, H., Banuls, AL., Ouedraogo, R. and Godreuil, S. (2017). Emergence et diffusion de la resistance aux antibiotiques en Afrique de l’Ouest : facteurs favorisants et evaluation de la menace. Med Sante Trop. 27 : 147-154. doi : 10.1684/mst.2017.0678
11. Keeley, AJ. and Nsutebu, E. (2021). Improving sepsis care in Africa: an opportunity for change. Pan Afr. Med. J. 40 (204). 10116004. doi: 10.11604/pamj.2021.40.204.30127.
12. Jacobs, J., Hardy, L., Semret, M., Lunguya, O., Phe, T., Affolabi, D. et al. (2019). Diagnostic Bacteriology in District Hospitals in Sub-Saharan Africa: At the Forefront of the Containment of Antimicrobial Resistance. Front Med (Lausanne); 6: 205. https://doi.org/10.3389/fmed.2019.00205
13. Lim, R., Ellett, LK., Roughead, EE., Cheah, PY. and Masnoon, N. (2021). Correction: Anastopoulos et al. Multi-Drug Featurization and Deep Learning Improve Patient-Specific Predictions of Adverse Events. Int. J. Environ. Res. Public Health .19(7):4216. doi: 10.3390/ijerph19074216.
14. Legba, B.B., Dougnon, V., Koudokpon, H., Mero, S., Elovainio, R., Parry, M., et al. (2023) Assessment of blood cultures and antibiotic susceptibility testing for bacterial sepsis diagnosis and utilization of results by clinicians in Benin: A qualitative study. Front. Public Health. 10:1088590. doi: 10.3389/fpubh.2022.1088590
15. Maraolo, AE., Ong, DSY., Cimen, C., Howard, P., Kofteridis, DP., Schouten, J., et al. (2019). Organization and training at national level of antimicrobial stewardship and infection control activities in Europe: an ESCMID cross-sectional survey. Eur J Clin Microbiol Infect Dis. 38(11):2061-2068 doi 10.1007/s10096-019-03648-2
16. Monnier, E-C., Avry, S., El-Hamamsy, L., Pulfrey, C., Caneva, C., Mondada, F., et al. (2023). From teacher to teacher-trainer: A qualitative study exploring factors contributing to a successful train-the-trainer digital education program. Social Sciences & Humanities Open. 8:100518. https://doi.org/10.1016/j.ssaho.2023.100518
17. Oladeinde, BH., Omoregie, R., Odia, I., and Osakue, EO. (2014). Continuous professional training of medical laboratory scientists in Benin City, Nigeria. Afr. J. Health Prof. Educ. 6:60–63.
18. Abrudan, M., Matimba, A., Nikolic, D., Hughes, D., Argimón, S., Kekre, M., et al. (2021). For the NIHR Global Health Research Unit on Genomic Surveillance of Antimicrobial Resistance. Train-the-Trainer as an Effective Approach to Building Global Networks of Experts in Genomic Surveillance of Antimicrobial Resistance (AMR). Clin. Infect. Dis. 73(4): 283 -– 289
19. Mormina, M. and Pinder, S. (2018). A conceptual framework for training of trainers (ToT) inventions in global health. Glob. Health. 14: 100.
20. Snowden, B., Lahiri, S., Dutton, R. and Morton, L. (2022). Achieving and Sustaining Change Through Capacity Building Train-the-Trainer Health Initiatives in Low-and Middle-Income Countries: A Systematic Review. J Contin Educ Health Prof. 43(2):96-103. doi: 10.1097/CEH.0000000000000458
21. Ministry of Health Benin. (2022). Multisectoral National Action Plan for the fight against Antimicrobial Resistance in the Republic of Benin (in French). 2nd ed. 98p95p
22. Biggs, J. (1996). Enhancing teaching through constructive alignment. High. Educ. 32: 347-364.
23. Kanselaar, G. (2002). Constructivism and socio-constructivism, https://kanselaar.net/wetenschap/files/Constructivism-gk.pdf
24. Campos-Zamora, M., Gilbert, H., Esparza-Perez, RI., Sanchez-Mendiola, M., Gardner, R., Richards, et al. (2022). Continuing professional development challenges in a rural setting: A mixed-methods study. Perspect Med Educ. 11:273-280. doi: 10.1007/s40037-022-00718-8.

**Figure 1**: Flow chart of the training course.

**Table 1**: Topics covered by the training course, detailed contents, and examples of implementation.

| **Topics** | **Content** | **Implementation examples** |
| --- | --- | --- |
| Conducting blood culture  (Days 1 and 2) | - Pre-training test - Taking a blood sample - Culture media bottles for blood culture - Culture conditions (temperature, incubation time, etc.) - Recognizing the growth in a bottle - Quality control - Presentation of the growth results | Searching for information on different techniques for blood sampling, attaining consensus on the best practice for blood culturing. Practicing sampling in pairs under nurse’s supervision, gram-staining directly from the culture bottle. |
| Bacterial identification (Day 3) | - Microscopic observation and gram- staining - Choice of solid culture media - Preparation of culture media - Seeding techniques - Description of colonies and other cultural characteristics of bacteria - Biochemical identification | Searching for information on biochemical tests for bacterial identification, assessing the results. Conducting the API 20E test in practice. |
| Conducting antibiotic susceptibility testing (AST)  (Day 4) | - Different methods for performing AST - Criteria for choosing the antibiotics to be tested - Incubation conditions (temperature, duration) - Interpretation of the results of the AST - Identification of ESBLs: microbiological and biochemical techniques - Quality control - Study of the EUCAST guidelines | Searching for information and discussing different ways to carry out AST, preparation of the inoculum, techniques of inoculation, sharing of experience between professionals. Conducting the laboratory work by the trainees. |
| Quality assurance in bacteriological laboratory  (Day 5) | - Quality criteria - Internal quality control - External quality assessment - Maintenance of the laboratory equipment - Quality control of laboratory reagents, culture media, antibiotic disks | Clarification of the concept of quality assurance in microbiology, conducting quality control on culture media, antibiotic discs and sterility and fertility testing of culture media. |
| Closing up  (Day 6) | - Post-training test - Joint discussion about the learnt skills - Collecting feedback about organization of the course - Closing ceremony and giving the certificates |  |

Table 3: The average pre-test and post-test scores for each group (maximum score 20).

|  | | Trainer group  (N=10) | Trainee group 1  (N=13) | Trainee group 2  (N=13) | Trainee group 3  (N=14) |
| --- | --- | --- | --- | --- | --- |
| Pre-test | score  (Mean**±SD)** | 11.0 **±** 3.7 | 9.6 **±** 2.4 | 9.8 **±** 3.4 | 10.3 **±** 4.2 |
|  | The range of scores | [4.50-16] | [4.50-13.75] | [5-14] | [5-15] |
| Post test | Post-test score  (Mean**±SD)** | 14.5 **±** 1.6 | 15.3 **±** 1.6 | 14.0 **±** 1.7 | 14.7 **±** 3.1 |
|  | The range of scores | [11-17] | [12-18.25] | [8-17] | [10-19] |
| Improvement average | | 3.5 | 5.7 | 4.2 | 4.4 |
| p-value | | 0,0012 | | | |

Table 2: Online material indicated to the participants before and during the training.

| Massive Open Online Courses (MOOCs) used as pre-assignment | - ***OpenWHO*** (Learning Platform of World Health Organization)   - Basic microbiology, https://openwho.org <https://openwho.org/courses/IPC-MICRO-EN>  - Antimicrobial stewardship: A competency-based approach, <https://openwho.org/courses/AMR-competency>   - ***Fleming Fund*** Online AMR Course, new modules <https://www.flemingfund.org/publications/fleming-fund-online-amr-course/> |
| --- | --- |
| Online audiovisual and reading materials utilized during the training | • Blood Culture Sampling: https://www.youtube.com/watch?v=ZZPhVSyWHNY&t=69s  • Hemoculture: https://www.youtube.com/watch?v=Z-2ejkgDgak&t=57s  • Prélèvement des hémocultures: https://www.youtube.com/watch?v=27FJSh8NAR4&t=66s  • Antimicrobial Susceptibility Testing and EUCAST Expert Update - Prof Jean Philippe Lavigne : https://www.youtube.com/watch?v=Efq-MSnp8Kw  • EUCAST videos (English)/Gunnar Kahlmeter : https://www.youtube.com/playlist?list=PLQU_kWRWBld4fDhv1T1KOR5bKUUTJ2v6W  • https://www.eucast.org/;  • https://www.labce.com/;  • https://amr-learninghub.org/; |

Note: The OpenWHO courses provide transcripts and audio-translation in several major languages, so the participants could use the language of their preference.
