# Supplementary material for: Strengthening clinical bacteriology laboratory diagnostics to combat sepsis and antimicrobial resistance in Benin: a train-the-trainer approach
Source: Front Med (Lausanne). 2024 Apr 19;11:1281418. doi: 10.3389/fmed.2024.1281418 (PMC11066218; doi:10.3389/fmed.2024.1281418)
Supplement: Supplementary file 2 [file Table_2.DOCX]

**Appendix**

**ASSESSMENT OF THE TRAINING BY THE PARTICIPANTS**

A number of achievements were noted by the participants. The main ones were improved knowledge and practical skills in blood sampling for blood cultures, choice of antibiotic discs, antibiotic susceptibility testing and the search for ESBLs using the double synergy of action test between amoxicillin + clavulanic acid and third generation cephalosporins. They will also be able to work on quality assurance in the bacteriology laboratory. The participants strongly recommended that a system be set up to monitor and supervise laboratories in general and trained technicians in particular, to ensure that what they learn in training is applied in the field. Some laboratories asked for help in calibrating their equipment and drafting procedures.

**PRE-TEST/POST-TEST**

**Theory test (The questions were presented to the students in French)**

1. What is sepsis?
2. What makes you suspect contamination of a blood culture sample?
3. In the laboratory, you were asked to analyze a blood culture sample. A strain of *Pseudomonas aeruginosa* was identified. Describe the major steps that led to this finding.
4. In the laboratory, you were asked to analyze a blood culture sample. A strain of *Streptococcus* was identified. Describe the major steps that led to this finding.
5. What are the criteria for choosing antibiotic discs for susceptibility testing?
6. How to perform bacteriological quality control for antibiotic discs and culture media?

**Practical Exam**

1. Choose one sample.
2. Perform macroscopic and microscopic examination of the sample.
3. Choose the right growing medium and inoculate the sample on it.
4. Justify your choice.

**COURSE MATERIAL**

The communications delivered during the training sessions, and the developed procedures, have been placed in a Google Drive folder and are accessible through the link below: <https://urlz.fr/oOno>
