# Supplementary material for: Strengthening clinical bacteriology laboratory diagnostics to combat sepsis and antimicrobial resistance in Benin: a train-the-trainer approach
Source: Front Med (Lausanne). 2024 Apr 19;11:1281418. doi: 10.3389/fmed.2024.1281418 (PMC11066218; doi:10.3389/fmed.2024.1281418)
Supplement: Supplementary file 3 [file Table_3.DOCX]

**Table 2**: Online material indicated to the participants before and during the training.

| Massive Open Online Courses (MOOCs) used as pre-assignment | - ***OpenWHO*** (Learning Platform of World Health Organization)   Basic microbiology, https://openwho.org <https://openwho.org/courses/IPC-MICRO-EN>  Antimicrobial stewardship: A competency-based approach, <https://openwho.org/courses/AMR-competency>   - ***Fleming Fund*** Online AMR Course, new modules <https://www.flemingfund.org/publications/fleming-fund-online-amr-course/> |
| --- | --- |
| Online audiovisual and reading materials utilized during the training | • Blood Culture Sampling: https://www.youtube.com/watch?v=ZZPhVSyWHNY&t=69s  • Hemoculture:  https://www.youtube.com/watch?v=Z-2ejkgDgak&t=57s  • Prélèvement des hémocultures: https://www.youtube.com/watch?v=27FJSh8NAR4&t=66s  • Antimicrobial Susceptibility Testing and EUCAST Expert Update - Prof Jean Philippe Lavigne : https://www.youtube.com/watch?v=Efq-MSnp8Kw  • EUCAST videos (English)/Gunnar Kahlmeter : https://www.youtube.com/playlist?list=PLQU_kWRWBld4fDhv1T1KOR5bKUUTJ2v6W  • https://www.eucast.org/  • https://www.labce.com/  • https://amr-learninghub.org/ |

Note: The OpenWHO courses provide transcripts and audio-translation in several major languages, so the participants could use the language of their preference.
